# Machine learning for predicting survival of colorectal cancer patients

**DOI:** 10.1038/s41598-023-35649-9

**Published:** 2023-06-01

**Authors:** Lucas Buk Cardoso, Vanderlei Cunha Parro, Stela Verzinhasse Peres, Maria Paula Curado, Gisele Aparecida Fernandes, Victor Wünsch Filho, Tatiana Natasha Toporcov

**Affiliations:** 1 Núcleo de Sistemas Eletrônicos Embarcados, Instituto Mauá de Tecnologia, São Paulo, 09580-900 Brazil; 2 Information and Epidemiology, Fundação Oncocentro de São Paulo, São Paulo, 05409-012 Brazil; 3 Epidemiology and Statistics on Cancer Group, A.C. Camargo Cancer Center, São Paulo, 01525-001 Brazil; 4 Epidemiology Department, Faculdade de Saude Pública da Universidade de São Paulo, São Paulo, 01246-904 Brazil

**Keywords:** Cancer, Gastrointestinal cancer

## Abstract

Colorectal cancer is one of the most incident types of cancer in the world, with almost 2 million new cases annually. In Brazil, the scenery is the same, around 41 thousand new cases were estimated in the last 3 years. This increase in cases further intensifies the interest and importance of studies related to the topic, especially using new approaches. The use of machine learning algorithms for cancer studies has grown in recent years, and they can provide important information to medicine, in addition to making predictions based on the data. In this study, five different classifications were performed, considering patients’ survival. Data were extracted from Hospital Based Cancer Registries of São Paulo, which is coordinated by Fundação Oncocentro de São Paulo, containing patients with colorectal cancer from São Paulo state, Brazil, treated between 2000 and 2021. The machine learning models used provided us the predictions and the most important features for each one of the algorithms of the studies. Using part of the dataset to validate our models, the results of the predictors were around 77% of accuracy, with AUC close to 0.86, and the most important column was the clinical staging in all of them.

## Introduction

The analysis of the survival of cancer patients is fundamental for the planning and evaluation of health services. Additionally, the identification and validation of prognostic factors are important to guide the treatment protocol.

Epidemiological studies have used statistical models, based on pre-established predictors for the prognosis of survival in patients with colorectal cancer (CRC). Such techniques have limitations related to the adaptation of models, changes in the reality, and potential reduction in accuracy over time^1^. The most common statistical models are linear and depart from explicit descriptions of the relationships between data. Currently, artificial intelligence (AI) has been collaborating in the diagnosis of several diseases^2,3^ and in the evaluation of survival^4^, the machine learning technique, an application based on artificial intelligence data, in which systems learn and improve automatically without explicit programming^5^, has been used in the search for an evaluation that demands fewer human resources, possibly more accurate and perennial survival. They are quickly and easily adaptable to new realities and their use has been tested in cancer studies^6^.

Since models using machine learning do not provide structure and parameters in an explicit and easily interpretable way, it becomes crucial to test their use and their accuracy with real data. In recent years, cancer registry data, such as the US Surveillance, Epidemiology and End Results (SEER), have been used to predict mortality or survival in the US using artificial intelligence^5,7^.

Colorectal cancer (CRC) is among the ten most incidents in the world^8^. It is estimated that approximately 10% of cancer cases in the world in 2020 will be in the colon or rectum, corresponding to approximately 1.8 million new cases annually^9^, with an increasing trend in both genders. In Brazil, around 41 thousand new cases are estimated between 2020 and 2022^10^. The Hospital Based Cancer Registries of São Paulo state (RHC-SP), based at the Fundação Oncocentro do Estado de São Paulo (FOSP), covers a population of approximately 30 million inhabitants, with 33,000 cases of colorectal cancer, configuring a unique opportunity to carry out of mortality or survival prediction studies for Brazilian patients. The objective of the present study is to evaluate and compare the validity of three artificial intelligence algorithms for predicting the survival of patients with CRC treated in São Paulo, the most populous state in Brazil, from 2000 to 2021, based on data from the RHC-SP.

## Results

### Characteristics of the population

**Figure 1:**
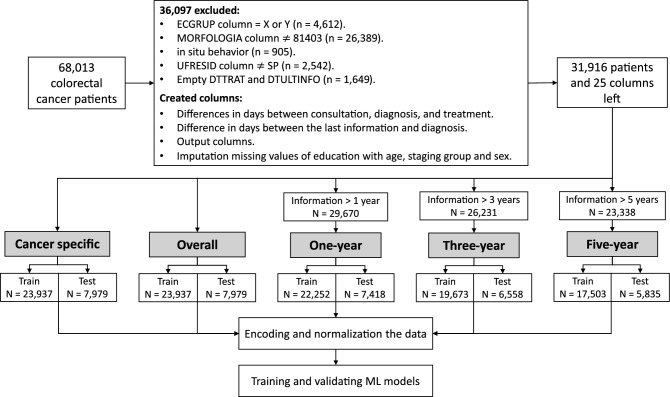
Diagram with the steps performed in the studies. All specific selections, created columns, and preprocessing steps are described, with a greater focus until the division of the data set into training and testing for each analysis. After this, we have the encoding of the features to numerical values and their normalization, followed by the steps of training and validation of the created models.

When analyzing the patients’ survival over the years after the diagnosis, extracting this information directly from the dataset, a decay can be seen up to the fifth year, being 77% of survival in 1 year, 59% in 3 years, and 53.2% in 5 years, Supplementary Fig. S1. The selection of data, creation of columns, and pre-processing steps that were used in the analyses are shown according to the driagram in Fig. 1, 31,916 patients were eligible. For the patients’ survival studies (1, 3, and 5 years), a selection was made to remove data from patients who were not followed up for the *label* period, so 29,670, 26,231, and 23,338 patients were eligible for the analyzes 1-year, 3-year, and 5-year survival, respectively.

### Training and validation

The division of the dataset into training and testing was performed randomly, with 75% for training and 25% for test data, in addition, there was no significant difference between the sets in the percentage of patients contained in each class, Table 1.

**Table 1:** Percentage of patients in each class for the training and test sets.

	Cancer specific (%)	Overall (%)	One-year (%)	Three-year (%)	Five-year (%)
Train					
No (Class 0) Yes (Class 1)	58.8 41.2	47.0 53.0	21.7 78.3	48.0 52.0	63.4 36.6
Test					
No (Class 0) Yes (Class 1)	59.0 41.0	47.2 52.8	22.1 77.9	48.0 52.0	63.8 36.2

There are no major differences in the distribution of classes between the training and testing sets in all analyses.

Models were trained and fitted for each analysis and validations were performed using the confusion matrix for the test data, along with the ROC curve and the corresponding training and testing AUC. For the *Random Forest* and *XGBoost* models, a search for the best hyperparameters of these models was also performed for each of the analyses, a summary of the results for the test set is presented in Table 2. Observing the AUC values, it is noticed that some of the models show a significant difference between the values for training and testing, especially the *Random Forest* models. This characterizes the problem of overfitting in these models, which occurred due to the search for parameters in a wider range of values. However, the *XGBoost* models did not show this problem so pronouncedly and obtained the best accuracy results in all studies.

**Table 2:** Summary of the results.

	Naive Bayes		Random Forest			XGBoost	
	Acc (%)	AUC	Acc (%)	AUC	Acc (%)		AUC
Cancer specific	50.2	0.765	76.8	0.844	77.1		0.845
Overall	50.0	0.781	77.2	0.852	77.7		0.857
One-year	62.3	0.772	76.8	0.842	77.4		0.846
Three-year	59.3	0.743	74.4	0.823	74.7		0.826
Five-year	50.1	0.759	77.1	0.853	77.9		0.858

Accuracy and AUC values for each of the three models used, using the test data.

Finally, some neural network possibilities were tested for these data, both sequential and more complex models, but the performance was lower than the machine learning algorithms used, obtaining lower accuracy than the *Random Forest* and *XGBoost* models in all tests performed. Therefore, the use of neural networks in this study was not further explored.

#### Cancer specific survival

**Figure 2:**
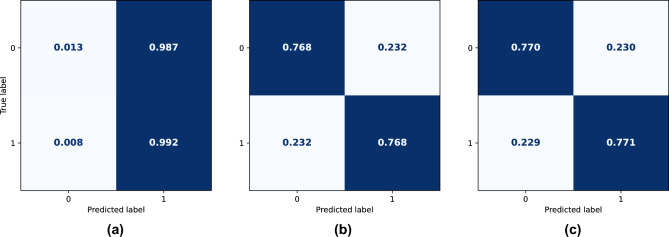
(**a**) Naive Bayes, (**b**) Random Forest, (**c**) XGBoost. Confusion matrices of the models, cancer specific survival. The Naive Bayes model (**a**) had the worse performance, besides not having balanced accuracy in both classes. On the other hand, the Random Forest (**b**) and XGBoost (**c**) models had a performance with a very similar accuracy.

**Figure 3:**
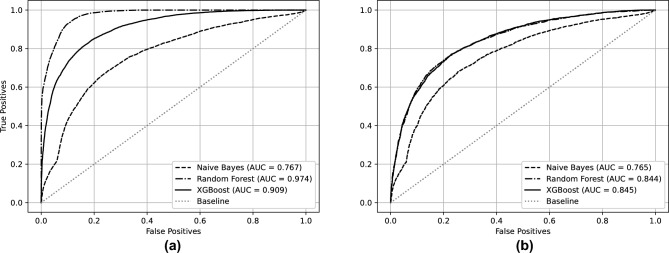
(**a**) Training, (**b**) Test. ROC curves of the models, cancer specific survival. As expected, the Naive Bayes model showed the worst AUC values, both for the training and test sets. Looking at the curves for the Random Forest and XGBoost models, it can be noticed that there is some overfitting in both models, especially in the case of the Random Forest model, due to the difference in AUC values between the training and test metrics.

The *Naive Bayes* model had the worst performance among those used, observing the accuracy of the model, we obtain an unbalanced classification, getting more data from class 1 to class 0 (Fig. 2a), in the ROC curve, the value was AUC = 0.767 for training and 0.765 for testing (Fig. 3). With the *Random Forest* model, the accuracy was almost 77% in both classes (Fig. 2b), the training and testing AUC values were 0.974 and 0.844, respectively (Fig. 3). Finally, using the *XGBoost* model, a better performance, comparing with *Random Forest* model was obtained, with more than 77% of accuracy in the prediction of both classes (Fig. 2c). AUC values were 0.909 for training and 0.845 for testing (Fig. 3).

**Figure 4:**
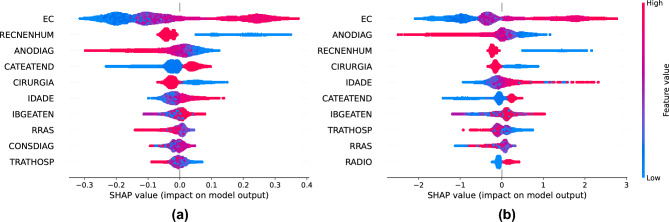
(**a**) Random Forest, (**b**) XGBoost. Feature importances of the models, cancer specific survival. The SHAP values show the most important features for the Random Forest and XGBoost models, allowing for analysis and validation, based on medical knowledge, of the algorithms’ training. Both presented similar columns among the top ten, with only the order varying, probably due to differences between the two algorithms.

The five most important *features* of the *Random Forest* model were clinical staging, presence of recurrence, year of diagnosis, service category and surgery (Fig. 4a). For the *feature* service category, the conclusions are similar to those of the clinical staging column, lower values have a negative impact and higher values have a positive impact. In the columns’ presence of recurrence, year of diagnosis, and surgery, higher values of these *features* negatively influence the prediction, contributing more to class 0, which means that the patient did not die from cancer. The lower values of the *features* contributed the most to class 1.

Regarding the *XGBoost* model, four of them are similar to the conclusions of the *Random Forest* model (Fig. 4b), they are clinical staging, year of diagnosis, presence of recurrence, and surgery. The other column that appeared was age, which had higher values having a greater impact on patient death (class 1) and lower values for survival (class 0).

#### Overall survival

The *Naive Bayes* model had the worst performance among those used, observing its accuracy, we obtain an unbalanced classification, so the model got more data from class 0 in relation to class 1 (see Supplementary Fig. S2a), in the ROC curve, the value was AUC = 0.773 for training and 0.781 for testing (Supplementary Fig. S3). With the *Random Forest* model, the accuracy was 77% in both classes (see Supplementary Fig. S2b), the training and test AUC values were 0.958 and 0.852, respectively (Supplementary Fig. S3). Finally, using the *XGBoost* model, the best performance was obtained, with almost 78% accuracy in class prediction (see Supplementary Fig. S2c). AUC values were 0.873 for training and 0.857 for testing (Supplementary Fig. S3).

The five most important *features* of the *Random Forest* model were clinical staging, year of diagnosis, age, presence of recurrence, and surgery (Supplementary Fig. S4a). For the *features* age the conclusions are similar to the staging column, smaller values have a negative impact and larger values have a positive impact. In the columns’ year of diagnosis, presence of recurrence, and surgery, higher values of these *features* negatively influence the prediction, contributing more to class 0, which means that the patient did not die for any reason. The lower values of the *features* contributed the most to class 1. Regarding the *XGBoost* model, all of them are similar to the conclusions of the *Random Forest* model (Supplementary Fig. S4b), they are clinical staging, year of diagnosis, age, presence of recurrence and surgery.

#### One-year survival

The *Naive Bayes* model had the worst performance among those used, observing the accuracy, the model predicts almost all data as being of class 0 (see Supplementary Fig. S5a), in the ROC curve, the value was AUC = 0.761 for training and 0.772 for testing (Supplementary Fig. S6). With the *Random Forest* model, the accuracy was 77% in both classes (see Supplementary Fig. S5b), the training and test AUC values were 0.862 and 0.842, respectively (Supplementary Fig. S6). Finally, using the *XGBoost* model, a performance a little bit higher than *Random Forest* was obtained, with more than 77% accuracy in the prediction (see Supplementary Fig. S5c). AUC values were 0.865 for training and 0.846 for testing (Supplementary Fig. S6).

The five most important *features* of the *Random Forest* model were clinical staging, in-hospital treatment, surgery, presence of recurrence, and chemotherapy (Supplementary Fig. S7a). For the *feature* presence of recurrence, the conclusions are similar to the staging column, higher values have a negative impact and lower values have a positive impact. In the in-hospital treatment, surgery, and chemotherapy columns, we have the opposite. Regarding the *XGBoost* model, the conclusions are similar to the *features* of the *Random Forest* model (Supplementary Fig. S7b), they are clinical staging, in-hospital treatment, surgery, chemotherapy, and presence of recurrence.

#### Three-year survival

The *Naive Bayes* model had the worst performance among those used, observing the accuracy, almost all the predictions were for class 0, thus, the model was correct with few data from class 1 (see Supplementary Fig. S8a), in the ROC curve, the value was AUC = 0.756 for training and 0.743 testing (Supplementary Fig. S9). With the *Random Forest* model, the accuracy was more than 74% in both classes (see Supplementary Fig. S8b), the training and test AUC values were 0.953 and 0.823, respectively (Supplementary Fig. S9). Finally, using the *XGBoost* model, a better performance was obtained, with almost 75% accuracy in the prediction of both classes (see Supplementary Fig. S8c). AUC values were 0.895 for training and 0.826 for testing (Supplementary Fig. S9).

The five most important *features* of the *Random Forest* model were clinical staging, surgery, age, in-hospital treatment, and year of diagnosis (Supplementary Fig. S10a). For the *features* age and year of diagnosis, the conclusions are similar to those of the staging column, higher values have a negative impact and lower values have a positive impact. In the surgery, and in-hospital treatment columns, we have the opposite.

Regarding the *XGBoost* model, all of them are similar to the conclusions of the *Random Forest* model (Supplementary Fig. S10b), they are clinical staging, year of diagnosis, surgery, age, and in-hospital treatment.

#### Five-year survival

The *Naive Bayes* model had the worst performance among those used, the accuracy was unbalanced, with almost all the predictions being for class 1 (see Supplementary Fig. S11a), in the ROC curve, the value was AUC = 0.757 for training and 0.759 for testing (Supplementary Fig. S12). With the *Random Forest* model, the accuracy was 77% in both classes (see Supplementary Fig. S11b), the training and testing AUC values were 0.969 and 0.853, respectively (Supplementary Fig. S12). Finally, using *XGBoost*, the best performance was obtained, with almost 78% accuracy in class prediction (see Supplementary Fig. S11c). AUC values were 0.882 for training and 0.858 for testing (Supplementary Fig. S12).

The five most important *features* of the *Random Forest* model were clinical staging, year of diagnosis, surgery, age, and in-hospital treatment (Supplementary Fig. S13a). For the *features* year of diagnosis and age, the conclusions are similar to those of the staging column, higher values have a negative impact and lower values have a positive impact. In the surgery and in-hospital treatment columns, higher values positively influence the prediction, contributing more to class 1, which means that the patient survived the fifth year. The lowest values of these *features* contributed more to class 0.

About the *XGBoost* model, all of them are similar to the conclusions of the *Random Forest* model (Supplementary Fig. S13b), they are clinical stage, year of diagnosis, surgery, age, and in-hospital treatment.

## Discussion

Our study is one of the first to predict the survival of cancer patients in a large database using AI, and to verify the validity of these models in Brazil. The algorithm with the best survival prediction was *XGBoost*, followed by *Random Forest* and *Naive Bayes*. In all algorithms evaluated, both for overall and specific survival, the most impacting variable was clinical stage. The variables that best predicted survival in the best model were clinical stage, surgery performed, in-hospital treatment, age, and year of diagnosis.

In all survival analyzes, the advanced clinical staging was more decisive for the death prediction, a fact expected in survival analyses and repeatedly reported in the scientific literature by other authors. The survival values found considering the total number of patients, 77%, 59%, and 53.2% for 1, 3, and 5-year survival, is a little lower than the survival found for tumors considered to have regional metastasis in developed countries whose data are available at the global cancer observatory^11^. The importance of the in-hospital treatment and the year of diagnosis highlights the possibility of influence of contextual factors on patient survival, indicating possible inequalities related to the capacity of health services, available resources, and qualification of human resources of centers for the specific treatment of cancer.

The use of artificial intelligence to predict survival is a current topic in the scientific literature. Recently, Jiang et al.^7^ found over 90% accuracy for 5-year osteosarcoma survival in the US using the *XGBoost* algorithm. In our study, the model with the best accuracy (*XGBoost*) had lower sensitivity and specificity but was comparable to studies that used cancer registry data. Changee Lee et al.^5^ compared the accuracy of statistical models used for prediction with artificial intelligence algorithms to predict mortality from non-metastatic prostate cancer in the USA. Leonard et al.^12^ compared the survival prediction of patients with resected colon cancer using machine learning models and commonly used regression models. The authors verified a similarity between the accuracy of the AI prediction using only clinical and epidemiological data with that of other models considered the most accurate to date. In our study, we only compared AI models. This finding allows further studies comparing the best AI model with other statistical models.

A strength of our study is using data from almost all cancer centers of the State of São Paulo, what reduces the probability of selection bias. A potential limitation of our research refers to the models used, which do not allow the inclusion of patients lost to follow-up. However, given the low percentage of these cases (7%, 17.8%, and 26.9% for 1, 3, and 5-year survival, respectively), it was found that there was no significant change in the results when not including these patients in the training of the models. The survival found in our study is also similar to that of other studies that used survival methods that consider the follow-up time of those lost to follow-up, such as the Kaplan Meier, with the advantage of the study allowing the prediction of survival from the variables found in the model, and not just measure it. In conditions of disruption of health services, for example, the prediction of survival can be very useful to estimate the potential loss of survival.

In conclusion, our results showed that AI models proved to be valid for predicting the patients’ survival with colorectal cancer from hospital-based cancer registry data in low and middle-income countries, with emphasis on *XGBoost*. More studies are needed to compare the performance of AI models with the most common statistical models for prediction.

## Methods

### Study population

Patients treated between 2000 and 2021, residing in São Paulo state, were evaluated, totaling 31,916 cases of both genders, diagnosed with colorectal adenocarcinoma ([topography C18-C20, morphology 8140/3]; CID-O 3ed.). Data were extracted from the Deputy Directorate of Information and Epidemiology of Fundação Oncocentro de São Paulo^13^, coordinator of the RHC-SP, where we have information from more than 70 hospitals, public and private, from the state of São Paulo.

### Selection of variables

For survival analysis (1, 3, and 5 years), patients with the time between diagnosis and last information greater or equal than the survival time of the analysis, or who died in the period, were eligible. For example, in the 3-year survival analysis, patients with at least 3 years of treatment information and who died within the first 3 years after diagnosis were selected.

The variables alive in the first year, alive in the third year, alive in the fifth year, death by cancer and all-cause mortality are the labels of the respective analyses, so only the column that is used as the output of the classifiers will be left. The result was twenty-five columns for *features* and one *label* in all performed analyzes, the description of the *features* is presented in the Table 3.

**Table 3:** Description of features used in all studies.

Feature	Description
IDADE	Age of the patient
SEXO	Gender of the patient
IBGE	City code of patient’s residence according to IBGE with check digit
CATEATEND	Category of care at diagnosis
DIAGPREV	Previous diagnosis and treatment
EC	Clinical stage
TRATHOSP	Code of combination of treatments performed at the hospital
NENHUM	Treatment received at the hospital = none
CIRURGIA	Treatment received at the hospital = surgery
RADIO	Treatment received at the hospital = radiotherapy
QUIMIO	Treatment received at the hospital = chemotherapy
HORMONIO	Treatment received at the hospital = hormone therapy
TMO	Treatment received at the hospital = bone marrow transplant
IMUNO	Treatment received at the hospital = immunotherapy
OUTROS	Treatment received at the hospital = others
NENHUMANT	Treatment received outside the hospital and before admission = none
CONSDIAG	Difference in days between the consultation and diagnosis dates
TRATCONS	Difference in days between consultation and treatment dates
DIAGTRAT	Difference in days between treatment and diagnosis dates
ANODIAG	Year of diagnosis
DRS	Regional department of health
RRAS	Regionalized healthcare networks
RECNENHUM	No presence recurrence
IBGEATEN	IBGE code of the healthcare institution where the patient was treated
ESCOLARI2	Code for patient’s education level, with missing values filled

### Statistical analysis

The chi-square test was used to obtain the *p-values* for the variables used as *features* in the classification models. This test was chosen because there are no continuous columns in the dataset, so it is possible to use chi-square in all the *features*.

The *p-values* corresponding to each column of the dataset are shown in Table 4. There are many null values, most of which are very low values approximated by zero, showing a relationship between the *features* and the *labels* under analysis. Bigger values (close to one), show that there is no relationship between the column and the analyzed *label*.

**Table 4:** *p-values* of the columns used in the models, based on the chi-square test.

p-value	Cancer specific	Overall	One-year	Three-year	Five-year
IDADE	2.06 e−1	0.00	0.00	0.00	0.00
SEXO	7.10 e−6	0.00	1.85 e−2	3.36 e−9	5.77 e−9
IBGE	4.43 e−2	1.61 e−2	3.96 e−3	9.95 e−1	1.00
CATEATEND	0.00	0.00	0.00	0.00	0.00
DIAGPREV	0.00	0.00	5.36 e−8	2.21 e−1	0.00
EC	0.00	0.00	0.00	0.00	0.00
TRATHOSP	0.00	0.00	0.00	0.00	0.00
NENHUM	1.75 e−3	2.86 e−3	8.92 e−6	7.82 e−9	2.41 e−5
CIRURGIA	0.00	0.00	0.00	0.00	0.00
RADIO	0.00	0.00	0.00	0.00	2.19 e−1
QUIMIO	0.00	0.00	0.00	7.84 e−9	2.17 e−3
HORMONIO	8.09 e−1	8.64 e−1	4.11 e−9	7.35 e−10	1.77 e−7
TMO	7.05 e−1	6.41 e−1	8.29 e−1	5.12 e−1	2.53 e−1
IMUNO	7.39 e−1	9.60 e−1	6.87 e−1	1.23 e−1	3.21 e−2
OUTROS	0.00	7.38 e−1	6.25 e−6	0.00	0.00
NENHUMANT	7.48 e−1	2.77 e−1	9.62 e−1	9.53 e−1	6.53 e−1
CONSDIAG	1.00	1.00	1.00	1.00	1.00
TRATCONS	1.00	1.00	1.00	1.00	1.00
DIAGTRAT	1.00	1.00	1.00	1.00	1.00
ANODIAG	0.00	0.00	0.00	0.00	0.00
DRS	0.00	0.00	0.00	0.00	0.00
RRAS	0.00	0.00	0.00	0.00	0.00
RECNENHUM	0.00	0.00	0.00	0.00	3.45 e−6
IBGEATEN	0.00	0.00	0.00	0.00	0.00
ESCOLARI2	0.00	0.00	0.00	3.75 e−1	9.94 e−1

The independence of the input variables with the output of each of the studies was analyzed using the chi-squared test, and all the shown features were included in the analyses conducted.

### Construction of the models

The models were built based on the classifiers *Naive Bayes*^14^, *Random Forest*^14^ and *XGBoost*^15^. The choice of these three models was based on the differences between the algorithms of the models, with *Naive Bayes* being the most elementary, based solely on probabilities. The *Random Forest* and *XGBoost* algorithms, on the other hand, are more complex and showed good results in initial tests conducted by us. Both are based on decision tree concepts, with the difference that the former trains multiple trees in parallel and the latter processes in series, with a greater focus on correcting the wrong predictions of the previous tree.

The training is performed with the training set and the validation with the test set. The output variables were generated before training the models, being death by cancer (0 = survival and 1 = death by cancer), all-cause mortality (0 = survival and 1 = death by any reason), alive in the first year (0 = death in less than 1 year and 1 = survival), alive in the third year (0 = death in less than 3 years and 1 = survival), and alive in the fifth year (0 = death in less than 5 years and 1 = survival). All categorical variables were converted to numeric values. After that, all variables were normalized to have mean zero and variance one.

The *Naive Bayes* is a statistical model based on Bayes’ Theorem and uses as a principle the independence between the variables of the problem^16^. *Random Forest* uses several decision trees to perform the classification with the *bagging* method, which uses the random selection of *features* and voting to combine the results of the parallel trees and generate the classification^17^. *XGBoost* combines the methods *bagging* and *boosting*, the latter uses decision tree classifiers in series, so each subsequent tree is trained using the errors of the previous one, ultimately forming a model stronger and more accurate for classification^18^.

The three models have a binary output, 0 and 1, which represents that the probability of survival of the patient, depending on the output under analysis, is lower or greater than 0.5 and were used to obtain a comparison between different approaches in training.

The validation was done with the confusion matrix of the test set, to verify the performance and generalization of the models in each prediction class. The ROC curves were constructed for the training and test sets, using the AUC metric to evaluate the performance of the models.

### Ethical considerations

Following the Lei Geral de Proteção de Dados Pessoais (LGPD) of Law No. 13,709, August 14, 2018, Section II—Processing of Sensitive Personal Data, as it is a search with a secondary database, of public access, not containing personal data of the patients, the opinion of the Research Ethics Committee was waived.

### Supplementary Information

Supplementary Information.

## Data Availability

The raw database, with all types of cancer, are available in the FOSP website. The datasets generated and analysed, and the notebooks developed during the current study are available in the GitHub repository.

## References

[1] Kourou K, Exarchos TP, Exarchos KP, Karamouzis MV, Fotiadis DI (2015). Machine learning applications in cancer prognosis and prediction. Comput. Struct. Biotechnol. J..

[2] Uddin S, Khan A, Hossain ME, Moni MA (2019). Comparing different supervised machine learning algorithms for disease prediction. BMC Med. Inform. Decis. Mak..

[3] Battineni G, Sagaro GG, Chinatalapudi N, Amenta F (2020). Applications of machine learning predictive models in the chronic disease diagnosis. J. Pers. Med..

[4] Silva G (2023). Machine learning for longitudinal mortality risk prediction in patients with malignant neoplasm in são paulo, brazil. Artif. Intell. Life Sci..

[5] Lee C (2021). Application of a novel machine learning framework for predicting non-metastatic prostate cancer-specific mortality in men using the surveillance, epidemiology, and end results (seer) database. Lancet Digit. Heal..

[6] Huang S, Yang J, Fong S, Zhao Q (2020). Artificial intelligence in cancer diagnosis and prognosis: Opportunities and challenges. Cancer Lett..

[7] Jiang J (2021). Predictive model for the 5-year survival status of osteosarcoma patients based on the seer database and xgboost algorithm. Sci. Rep..

[8] Weiderpass, E. & Stewart, B. W. World cancer report. *The Int. Agency for Res. on Cancer (IARC)* (2020). 39432694

[9] Sung H (2021). Global cancer statistics 2020: Globocan estimates of incidence and mortality worldwide for 36 cancers in 185 countries. CA.

[10] INCA. *Estimativa 2020: Incidência de Câncer no Brasil*. https://www.inca.gov.br/sites/ufu.sti.inca.local/files/media/document/estimativa-2020-incidencia-de-cancer-no-brasil.pdf (2019).

[11] IARC. *Global Cancer Observatory: Cancer Survival in High-Income Countries*. International Agency for Research on Cancer - World Health Organization. https://gco.iarc.fr/survival/survmark/.

[12] Leonard G (2022). Machine learning improves prediction over logistic regression on resected colon cancer patients. J. Surg. Res..

[13] FOSP. *Diretoria Adjunta de Informação e Epidemiologia: Banco de Dados do rhc*. https://fosp.saude.sp.gov.br/fosp/diretoria-adjunta-de-informacao-e-epidemiologia/rhc-registro-hospitalar-de-cancer/banco-de-dados-do-rhc/.

[14] Pedregosa F (2011). Scikit-learn: Machine learn-ing in python. J. Mach. Learn. Res..

[15] Chen, T. & Guestrin, C. Xgboost: A scalable tree boosting system. *22nd ACM SIGKDD Int. Conf. on Knowl. Discov. Data Min.* (2016).

[16] Raschka, S. Naive bayes and text classification i-introduction and theory. *arXiv preprint *arXiv:1410.5329 (2014).

[17] Denil, M., Matheson, D. & De Freitas, N. Narrowing the gap: Random forests in theory and in practice. In *International Conference on Machine Learning* 665–673 (PMLR, 2014).

[18] Agrawal, K. *Xgboost Classifier Algorithm in Machine Learning*. LinkedIn. https://www.linkedin.com/pulse/xgboost-classifier-algorithm-machine-learning-kavya-kumar.

