# Author Correction: Machine learning for predicting survival of colorectal cancer patients

**DOI:** 10.1038/s41598-023-43248-x

**Published:** 2023-09-25

**Authors:** Lucas Buk Cardoso, Vanderlei Cunha Parro, Stela Verzinhasse Peres, Maria Paula Curado, Gisele Aparecida Fernandes, Victor Wünsch Filho, Tatiana Natasha Toporcov

**Affiliations:** 1 Núcleo de Sistemas Eletrônicos Embarcados, Instituto Mauá de Tecnologia, São Paulo, 09580-900 Brazil; 2 Information and Epidemiology, Fundação Oncocentro de São Paulo, São Paulo, 05409-012 Brazil; 3 Epidemiology and Statistics on Cancer Group, A.C. Camargo Cancer Center, São Paulo, 01525-001 Brazil; 4 Epidemiology Department, Faculdade de Saude Pública da Universidade de São Paulo, São Paulo, 01246-904 Brazil

Correction to: *Scientific Reports* https://doi.org/10.1038/s41598-023-35649-9, published online 01 June 2023

The Funding section in the original version of this Article was omitted. The Funding section now reads:

“Fundação de Amparo à Pesquisa do Estado de São Paulo (FAPESP), Brazil. Process Number: 2021/11794-4”.

The original Article has been corrected.

