# Supplementary material for: Machine learning for predicting survival of colorectal cancer patients
Source: Sci Rep. 2023 Jun 1;13:8874. doi: 10.1038/s41598-023-35649-9 (PMC10235087; doi:10.1038/s41598-023-35649-9)
Supplement: Supplementary file 1 — Supplementary Information. [file 41598_2023_35649_MOESM1_ESM.pdf]

Machine Learning for predicting survival of colorectal cancer patients

Lucas Buk Cardoso[1,*,+], Vanderlei Cunha Parro[1,+], Stela Verzinhasse Peres[2], Maria Paula Curado[3], Gisele Aparecida Fernandes[3], Victor Wünsch Filho[2,4], and Tatiana Natasha Toporcov[4]

[1]Instituto Mauá de Tecnologia, Núcleo de Sistemas Eletrônicos Embarcados, São Paulo, 09580-900, Brazil
[2]Fundação Oncocentro de São Paulo, Information and Epidemiology, São Paulo, 05409-012, Brazil
[3]A.C. Camargo Cancer Center, Epidemiology and Statistics on Cancer Group, São Paulo, 01525-001, Brazil
[4]Faculdade de Saúde Pública da Universidade de São Paulo, Epidemiology Department, São Paulo, 01246-904, Brazil
[*]lucas.cardoso@maua.br
[+]these authors contributed equally to this work

ABSTRACT

Colorectal cancer is one of the most incident types of cancer in the world, with almost 2 million new cases annually. In Brazil, the scenery is the same, around 41 thousand new cases were estimated in the last three years. This increase in cases further intensifies the interest and importance of studies related to the topic, especially using new approaches. The use of machine learning algorithms for cancer studies has grown in recent years, and they can provide important information to medicine, in addition to making predictions based on the data. In this study, five different classifications were performed, considering patients' survival. Data were extracted from Hospital Based Cancer Registries of São Paulo, which is coordinated by Fundação Oncocentro de São Paulo, containing patients with colorectal cancer from São Paulo state, Brazil, treated between 2000 and 2021. The machine learning models used provided us the predictions and the most important features for each one of the algorithms of the studies. Using part of the dataset to validate our models, the results of the predictors were around 77% of accuracy, with AUC close to 0.88, and the most important column was the clinical staging in all of them.

Supplementary Material

Removed columns

The state of residence column was removed, because we selected only residents of São Paulo state, as well as state of birth and city because it was evaluated that these two have no impact on the analyses performed. The columns with topography and morphology descriptions have detailed the types of cancer, but a very specific selection was used, so there is no information with great impact in these columns, the same goes for the topography and group of topography columns. As single selections were made for the behavior (value 3) and morphology (value 81403), both were removed.

Regarding to the treatment, only in-hospital treatment column was used, removing non-treatment, treatment, and out-of-hospital treatment, the latter results in the removal of all columns related to out-of-hospital treatment after admission. Other columns removed in the pre-processing were metastasis 01, metastasis 02, metastasis 03, metastasis 04, recurrence 01, recurrence 02, recurrence 03, recurrence 04, classification T, N and M, local recurrence, regional recurrence, distant recurrence, and qualification, for some reasons, one of them is that some are empty, in addition to not having confidence about the filling of these columns.

Other columns removed from the database were about the difference, in days, between the dates, being the difference between the date of the anatomopathological examination and consultation, between treatment and diagnosis, and between treatment and consultation.

Three columns were created that are the difference between the date of the last information and the dates of consultation, diagnosis, and treatment. With the information from the last information column, the columns of all-cause mortality and death by cancer. As for the patient survival columns (one-year, three-year and five-year survival), the column with the last information was used together with the column on the difference between the last information and the diagnosis.

Subsequently, the age, clinical staging group, and sex columns were used to insert the values in the education variable that had this information ignored (value 9).

The age group columns were removed to perform the analyses, as it was decided to leave the column age of the patients.

Another four that were not considered are the difference between the date of the last information and the dates of consultation, diagnosis, and treatment, in addition to the last information, because they have data related to the *labels* used, the education was removed because we used the education column filled with age, sex and staging group. The staging group column was removed because it contains similar data to the clinical staging column, the latter having more information about the patients.

Supplementary images and results for the other studies

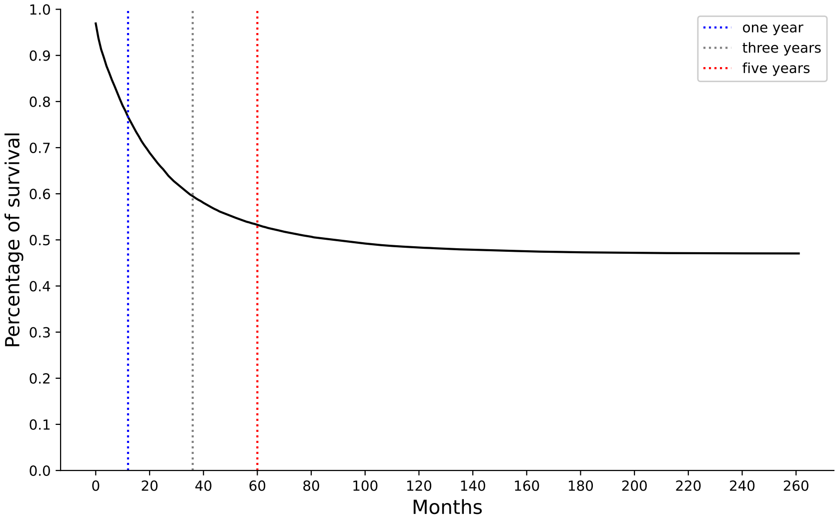

Supplementary Figure S1. Survival over 1, 3, and 5 years after diagnosis. It is possible to observe a decrease in survival as the months pass since the diagnosis, this information was extracted directly from the data.

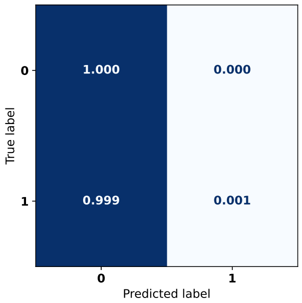

(a) Naive Bayes

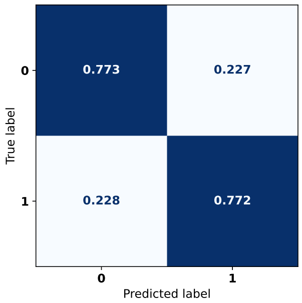

(b) Random Forest

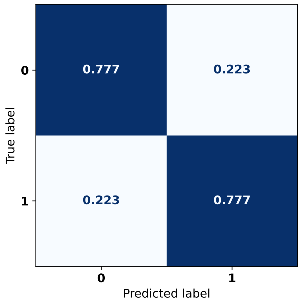

(c) XGBoost

Supplementary Figure S2. Confusion matrices of the models, overall survival. Naive Bayes model (a) had the worse performance, besides not having balanced accuracy in both classes. Random Forest (b) and XGBoost (c) models had a better performance with 77% and 78% of accuracy, respectively.

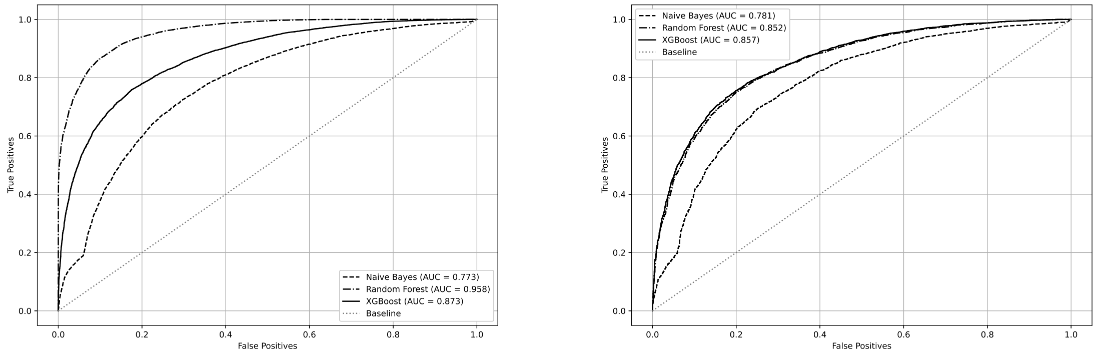

(a) Training **(b)** Test

Supplementary Figure S3. ROC curves of the models, overall survival. As expected, Naive Bayes showed the worst AUC values, for training and test sets. Looking at the curves for the Random Forest and XGBoost models, it can be noticed that there is some overfitting in both models, especially in the case of the Random Forest model, due to the difference in AUC values between the training and test.

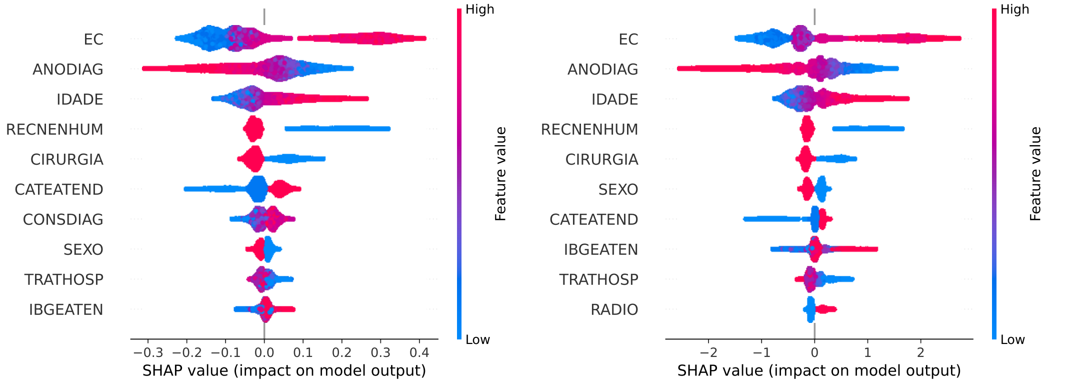

(a) Random Forest **(b)** XGBoost

Supplementary Figure S4. Feature importances of the models, overall survival. The SHAP values show the most important features for the Random Forest and XGBoost models, allowing for analysis and validation, based on medical knowledge, of the algorithms' training. Both presented similar columns among the top ten, with the order varying, probably due to differences between the two algorithms.

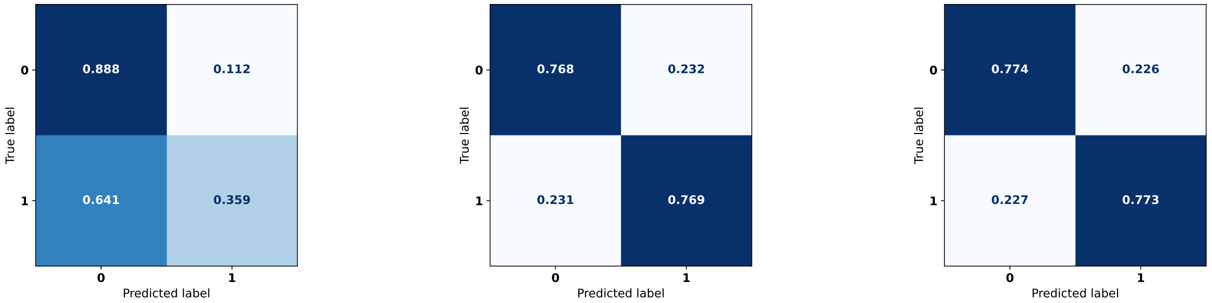

(a) Naive Bayes **(b)** Random Forest **(c)** XGBoost

Supplementary Figure S5. Confusion matrices of the models, one-year survival. Naive Bayes model (a) had the worse performance, besides not having balanced accuracy in both classes. On the other hand, the Random Forest (b) and XGBoost (c) models had a performance with 77% and 77.3% of accuracy, respectively.

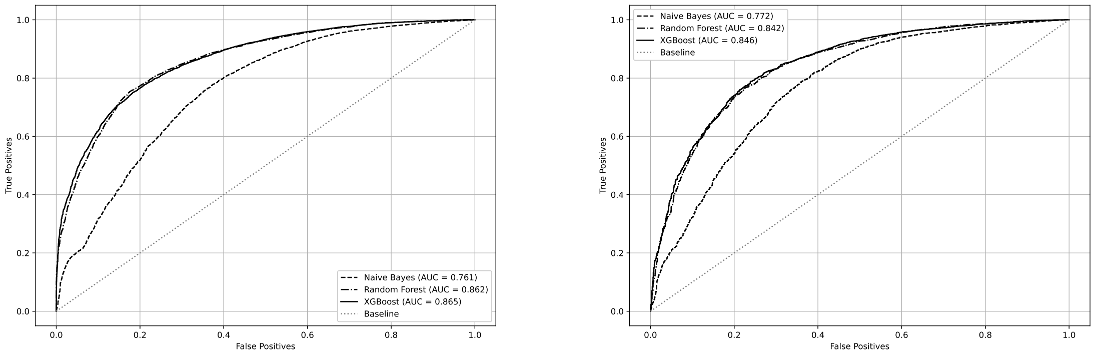

(a) Training **(b)** Test

Supplementary Figure S6. ROC curves of the models, one-year survival. As expected, Naive Bayes showed the worst AUC values, for training and test sets. Looking at the curves for the Random Forest and XGBoost models, here we do not have the overfitting problem as pronounced as in the other analyses, AUC values for the training and test sets of the models were close to each other.

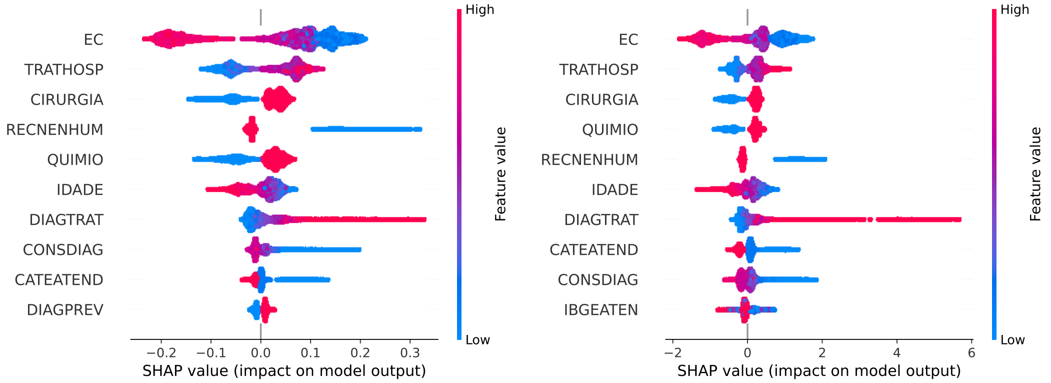

(a) Random Forest **(b)** XGBoost

Supplementary Figure S7. Feature importances of the models, one-year survival. The SHAP values show the most important features for the Random Forest and XGBoost models, allowing for analysis and validation, based on medical knowledge, of the algorithms' training. Both presented similar columns among the top ten, with the order varying, probably due to differences between the two algorithms.

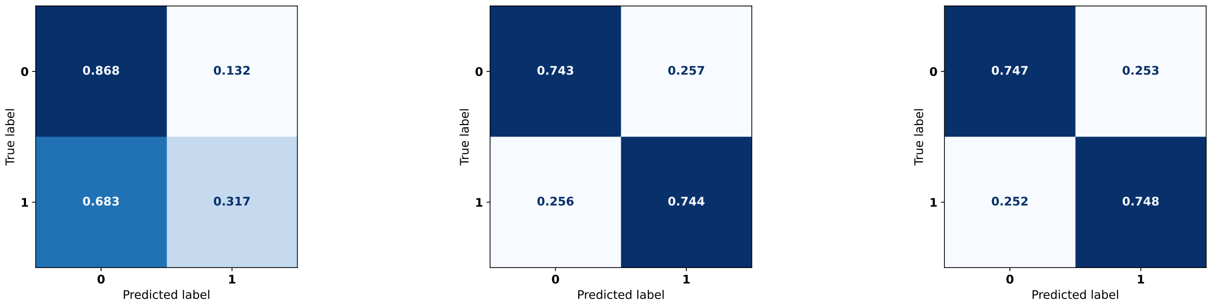

(a) Naive Bayes **(b)** Random Forest **(c)** XGBoost

Supplementary Figure S8. Confusion matrices of the models, three-year survival. Naive Bayes model (a) had the worse performance, because the model predicted almost all the test dataset as class 1. Random Forest (b) and XGBoost (c) models had a performance with more than 74% of accuracy for both models.

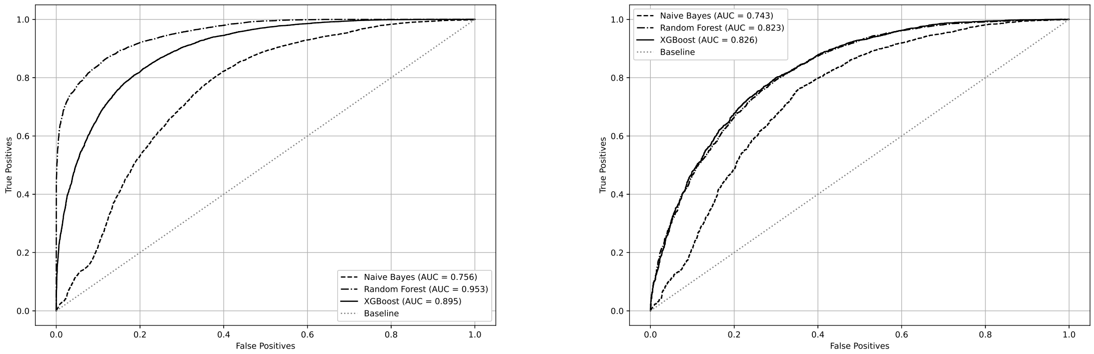

(a) Training

(b) Test

Supplementary Figure S9. ROC curves of the models, three-year survival. As expected, Naive Bayes showed the worst AUC values, for training and test sets. Looking at the curves for the Random Forest and XGBoost models, it can be noticed that there is some overfitting in both models, especially in the case of the Random Forest model, due to the difference in AUC values between the training and test.

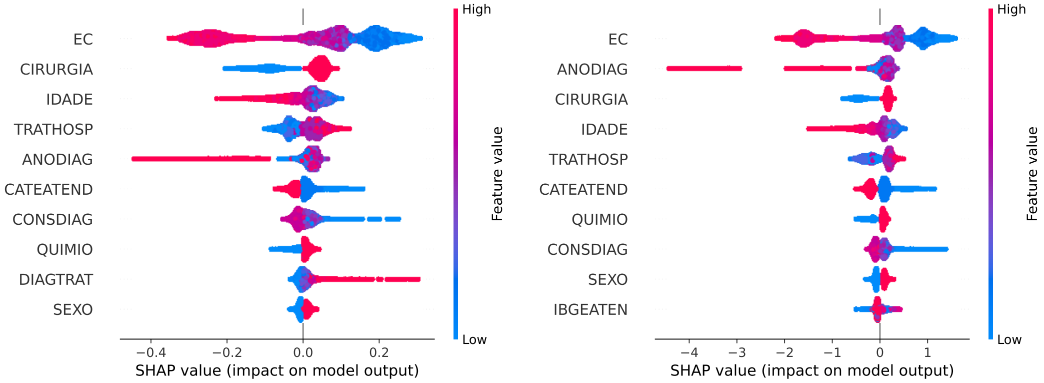

(a) Random Forest

(b) XGBoost

Supplementary Figure S10. Feature importances of the models, three-year survival. The SHAP values show the most important features for the Random Forest and XGBoost models, allowing for analysis and validation, based on medical knowledge, of the algorithms' training. Both presented similar columns among the top ten, with the order varying, probably due to differences between the two algorithms.

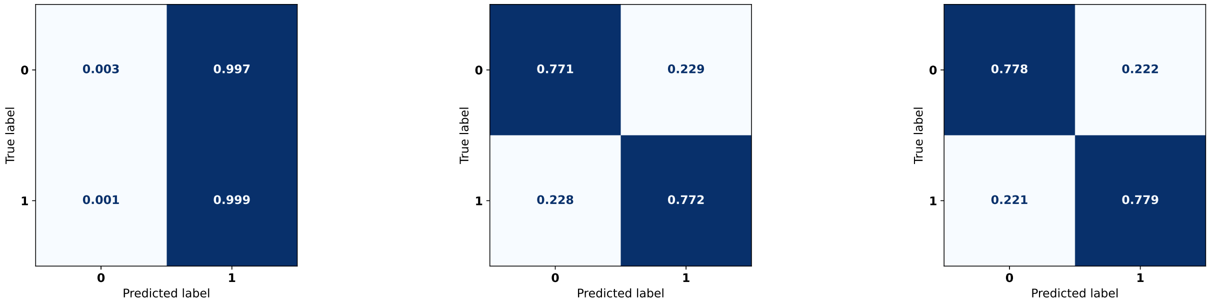

(a) Naive Bayes **(b)** Random Forest **(c)** XGBoost

Supplementary Figure S11. Confusion matrices of the models, five-year survival. Naive Bayes model (a) had the worse performance, besides not having balanced accuracy in both classes. On the other hand, the Random Forest (b) and XGBoost (c) models had a performance with 77% and 78% of accuracy, respectively.

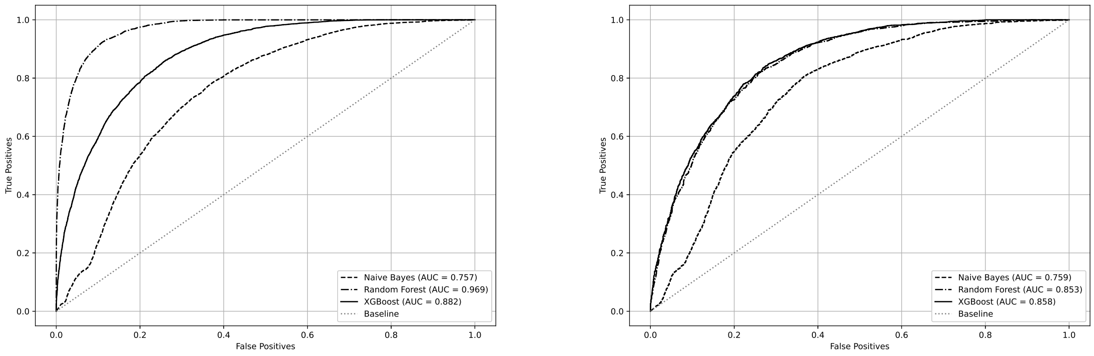

(a) Training **(b)** Test

Supplementary Figure S12. ROC curves of the models, five-year survival. As expected, Naive Bayes showed the worst AUC values, for training and test sets. Looking at the curves for the Random Forest and XGBoost models, it can be noticed that there is some overfitting in both models, especially in the case of the Random Forest model, due to the difference in AUC values between the training and test.

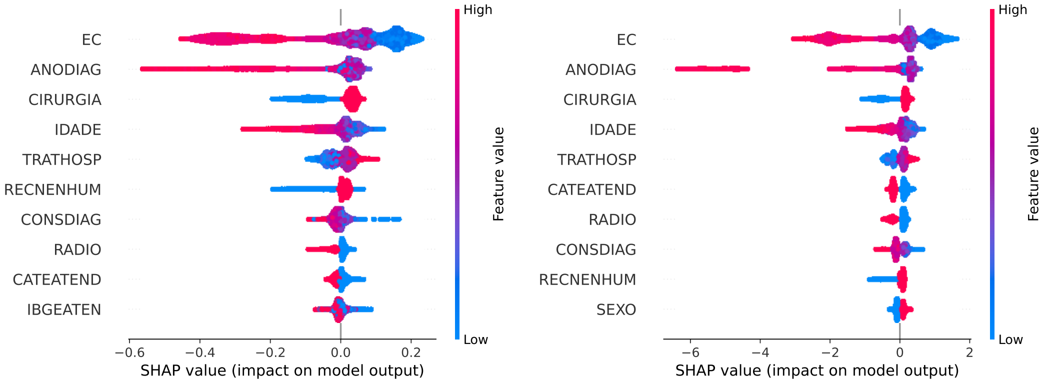

(a) Random Forest

(b) XGBoost

Supplementary Figure S13. Feature importances of the models, five-year survival. The SHAP values show the most important features for the Random Forest and XGBoost models, allowing for analysis and validation, based on medical knowledge, of the algorithms' training. Both presented similar columns among the top ten, with the order varying, probably due to differences between the two algorithms.
